# Impact of depression and anxiety on burden and management of episodic and chronic headaches – a cross-sectional multicentre study in eight Austrian headache centres

**DOI:** 10.1186/s10194-016-0603-3

**Published:** 2016-02-27

**Authors:** Karin Zebenholzer, Anita Lechner, Gregor Broessner, Christian Lampl, Gernot Luthringshausen, Albert Wuschitz, Sonja-Maria Obmann, Klaus Berek, Christian Wöber

**Affiliations:** Department of Neurology, Medical University of Vienna, Währinger Gürtel 18-20, 1090 Vienna, Austria; Department of Neurology, Medical University of Graz, Graz, Austria; Department of Neurology, Medical University of Innsbruck, Innsbruck, Austria; Headache Centre Seilerstätte, Hospital Sisters of Charity Linz, Linz, Austria; University Clinic of Neurology, Christian-Doppler-Klinik Salzburg, Salzburg, Austria; Neurological Office Vienna, Vienna, Austria; Department of Neurology, Klinikum Klagenfurt, Klagenfurt, Austria; Department of Neurology, aö. BKH Kufstein, Kufstein, Austria

**Keywords:** Episodic headache, Chronic headache, Migraine, Tension-type headache, Medication overuse headache, Depression, Anxiety, Psychiatric comorbidity, Burden, Quality of life

## Abstract

**Background:**

Recurrent and especially chronic headaches are associated with psychiatric comorbidities such as depression and anxiety. Only few studies examined the impact of depression and anxiety on episodic (EH) and chronic headache (CH), and data for Austria are missing at all. Therefore, the aim of the present study was to assess the impact of depression and anxiety on burden and management of EH and CH in patients from eight Austrian headache centres.

**Methods:**

We included 392 patients (84.1 % female, mean age 40.4 ± 14.0 years) who completed the Eurolight questionnaire. The treating physician recorded details about ever-before prophylactic medications. We used Hospital Anxiety and Depression Scale to assess depression and anxiety and compared patients with anxiety and/or depression to those without.

**Results:**

Depression and anxiety were more common in CH than in EH (64 % vs. 41 %, *p* < 0.0001). Presence compared to absence of depression and anxiety increased the prevalence of poor or very poor quality of life from 0.7 % to 13.1 % in EH and from 3.6 % to 40.3 % in CH (*p* = 0.001; *p* < 0.0001). Depression and anxiety had a statistically significant impact on employment status and on variables related to the burden of headache such as reduced earnings, being less successful in career, or feeling less understood. Neither in EH nor in CH health care use and the ever-before use of prophylactic medication was correlated with anxiety and/or depression.

**Conclusion:**

Depression and anxiety have a significant impact on quality of life and increase the burden in patients with EH and CH. Improved multidimensional treatment approaches are necessary to decrease disability on the personal, social and occupational level in these patients.

## Background

Headache is a frequent disorder with one-year prevalence rates of 10 – 18.6 % for migraine and 31 – 90 % for tension-type headache, and it is often interfering with everyday life [1–3]. Headache, especially migraine, is associated with comorbidities such as anxiety disorders and depression, ranging from 18 – 58 % for anxiety disorders and 17 – 47 % for depression [4–7]. In addition, headache patients, especially those with migraine or chronic daily headache, showed reduced quality of life [8, 9], and the burden caused by any headache is high with regard to lost workdays, lost days with household activities, lost family, social and leisure activities [10, 11]. Recent studies showed that significantly more patients with chronic migraine suffer from psychiatric comorbidities than patients with episodic migraine (31 – 58 % vs. 17 – 30 %), and that patients with chronic migraine had lower household incomes and were less likely to be employed fulltime [4, 5, 9].

To assess the prevalence of episodic headache (EH) and chronic headache (CH), management and burden of EH and CH in patients in Austrian headache centres, we performed a prospective, multicentre study in eight headache centres published in 2015 [11]. Within this study we collected also data on depression and anxiety. Depression and anxiety may cause additional burden in headache sufferers with respect to productivity loss, quality of life, health care utilization, and emotional burden. So far only few studies addressed the impact of psychiatric comorbidities in patients with migraine and headache in general [12–14]. None of these studies compared EH to CH and data for Austria are missing at all.

Therefore the aim of the present study was to assess the impact of depression and anxiety on burden and management of patients with EH and CH from eight Austrian headache centres.

## Methods

In April 2011 and September 2011 all consecutive patients attending one of eight Austrian headache centres for a first-time or follow-up visit were invited to participate in the study. Inclusion criteria were age ≥18 years, primary headache, medication overuse headache, and fluent German. Exclusion criteria were secondary headaches except medication overuse headache, fibromyalgia, other chronic pain disorders, and lacking knowledge of German. Four centres were at departments of medical universities, three centres were at large hospitals, and one centre was a large neurological office in Vienna. The study was approved by the ethics committee of the Medical Universities of Vienna, Graz, Innsbruck and Salzburg and local ethics committees of the other participating departments.

After giving written informed consent the patients completed the Eurolight questionnaire once. The Eurolight questionnaire was developed to gather data on the personal, social and economic impact of migraine, TTH and MOH in 15 countries in the European Union (EU) [10, 15]. It is a 103-item self-reporting questionnaire, validated in five languages, with good construct validity, good test-retest reliability and good internal consistency [15]. The questionnaire covers biographic data, headache symptoms, use of acute and prophylactic medications during the previous month, former examinations due to headache, quality of life as well as symptoms of anxiety and depression. The Eurolight questionnaire differentiates EH, i.e. headache on <15 days/month, and CH, i.e. headache on ≥15 days/month, and it is validated for diagnosing migraine, probable migraine, tension -type headache (TTH), probable tension-type headache (pTTH) and probable medication overuse headache (pMOH) according to ICHD-2 [15, 16]. Other headaches than mentioned above are classified as “other headache”. It does not allow diagnosing the underlying headache in patients with CH and pMOH. Therefore we diagnosed these patients based on their questionnaire entries concerning their most bothersome headache.

The questionnaire also includes the HALT index (Headache-attributed lost time index) for assessing days lost completely or partially because of headache [10, 17], the WHOQoL-8 (WHO quality of life assessment) [18], and the Hospital Anxiety and Depression scale (HADS) [19]. The HALT index captures in five questions the days lost completely or partially because of headache in the preceding three months and covers professional work, household activities or chores, and family, social or leisure activities. To estimate the burden of headache, and because data were not normally distributed, we summarized these lost days during the previous three months and categorized the impact of headache into 0-5 days lost, 6-10 days lost, 11-20 days lost, and >20 days lost. We assessed the patients’ quality of life by using the first question of the WHOQoL-8 that asks the patients to rate their general quality of life on a five-point scale (poor, very poor, neither good nor poor, good, very good) [18], and we used the HADS for assessing anxiety and depression [19]. The HADS has seven items each for assessing anxiety and depression. Anxiety or depression was rated as present if the HADS-A score or the HADS-D score was ≥8 [19]. If one of the subscales indicated depression or anxiety and the other subscale was missing, we rated depression and/or anxiety as present. If one subscale was not indicative of anxiety or depression and the other subscale was missing, we rated depression and anxiety data as missing.

In addition to the questionnaire completed by the patients, the treating neurologist filled in a questionnaire covering the clinical headache diagnosis and ever-before intake of five classes of standard prophylactic medication (betablocker, flunarizine, valproate, topiramate, amitriptyline). For each of these drug classes the treating neurologist had to assess, if it was contraindicated and if it was ever taken by the patient. If it had ever been taken, four additional questions had to be answered: (1) Was the treatment stopped because of intolerable adverse effects, (2) was the dose sufficient according to national and international guidelines [20, 21], (3) was the drug taken for at least three months, and (4) was headache frequency decreased by at least 50 % while taking this prophylactic drug?

For the initial study we screened 598 consecutive patients, 121 patients denied participation or had to be excluded, mainly because of lacking fluency in German, 36 questionnaires had to be excluded for incomplete data. Thus the analysis was based on 441 patients [11], of these 56.3 % had EH, 38.3 % had CH, and patients with CH had a significantly lower quality of life and significantly more often depression or anxiety [11]. For the present study we included all patients with available data on headache frequency in the Eurolight questionnaire and on prophylactic medication in the questionnaire completed by the treating neurologist.

### Statistics

We applied the standard computerized algorithm to analyse data derived from the Eurolight questionnaire [22]. We compared patients with EH to patients with CH, and for assessing the impact of depression and anxiety we compared patients with depression and/or anxiety and without depression and/or anxiety both in patients with EH and CH. We used numbers and percentages for descriptive statistics and Chi^2^-tests for comparing patients with and without depression and/or anxiety differentiating EH and CH. For comparisons of continuous variables we used t-tests. We calculated odds ratios to assess the risk of burden for presence versus absence of depression and/or anxiety in the entire group of patients and in those with EH and CH. For assessing the impact of depression and anxiety on the use of five classes of standard prophylactic medications we dichotomized ever-before use, contraindications, adverse effects, use for at least three months, intake of an adequate dose, and decrease in headache frequency by at least 50 % and compared presence in none versus presence in one or more of the drugs. Two-sided *p*-values < 0.05 were considered as statistically significant. As this was an exploratory study, we did not correct for multiple testing. Standardized Eurolight analyses were done with SAS 9.2, all other statistical analyses were performed using SPSS 20.0.

## Results

We included 392 patients with available data on headache frequency in the Eurolight questionnaire and with the completed additional questionnaire filled-in by the treating neurologist. Table 1 shows gender, depression and/or anxiety and headache diagnoses in EH compared to CH. Of 392 patients 301 (79.1 %) were female, 82 (20.9 %) male. The proportion of males was significantly higher among patients with CH (Table 1). Two-hundred-thirty-two patients (59.2 %) had EH, and 160 (40.8 %) patients had CH, among the latter 65 (40.6 %) had pMOH. Patients with EH and CH did not differ in age (40.3 ± 13.3 vs. 40.4 ± 14.9 years, *p* = 0.9). In EH 92 patients (40.2 %) and in CH 97 patients (63.8 %) had depression and anxiety or at least one of these psychiatric disorders (*p* < 0.0001). The prevalence of depression alone and anxiety alone was significantly higher in patients with CH (depression 43.6 %, anxiety 53.9 %) than in patients with EH (depression 22.8 %, anxiety 34.1 % *p* < 0.0001 (Table 1). Regarding headache diagnoses 199 (85.8 %) patients with EH had migraine; among the patients with CH 69 (43.1 %) had migraine, and 65 (40.6 %) had pMOH (Table 1).

**Table 1:** Gender, depression, anxiety and headache diagnoses in patients with EH and CH

	EH (*n* = 232)		CH (*n* = 160)			
	Patients		Patients		Chi^2^	p
	*n*	%	*n*	%		
Sex						
Female	198	84.1	112	71.9	13.5	<0.0001
Male	34	15.9	48	28.1		
Anxiety						
Missing data: 9						
Yes	78	34.1	83	53.9	14.9	<0.0001
No	151	65.9	71	46.1		
Depression						
Missing data: 19						
Yes	51	22.8	65	43.6	18.2	<0.0001
No	173	77.2	84	56.4		
Anxiety and/or depression						
Missing data: 14						
Yes	92	40.7	97	63.8	19.4	<0.0001
No	134	59.3	55	36.2		
Headache diagnosis						
Migraine	199	85.8	69	43.1	79.7	<0.0001
Tension-type HA	27	11.6	22	13.8		ns
Other HA	6	2.6	4	2.5		ns
pMOH	-	-	65	40.6		na

*EH* episodic headache, *CH* chronic headache, *pMOH* probable medication overuse headache, *ns* not significant; *na* not applicable

Looking at EH and CH patients separately, the mean age did not differ between with and without depression and/or anxiety: EH with depression and/or anxiety 39.7 ± 12.3 years, EH without depression and/or anxiety 41.2 ± 14.6 years; CH with depression and/or anxiety 38.8 ± 14.3 years, CH without depression and/or anxiety 41.6 ± 15.6 years. Also gender did not differ significantly between patients with and without depression and/or anxiety (Table 2). Table 2 shows the impact of anxiety and depression on patients with EH and CH. Headache diagnoses did not depend on presence or absence of anxiety and/or depression. Both in EH and CH significantly more patients with depression and/or anxiety had a worse quality of life (poor or very poor quality of life in EH 12 patients (13.1 %), in CH 30 patients (31.3 %), *p* < 0.0001), higher unemployment rates (in EH 23 patients (26.4 %), in CH 42 patients (45.2 %), *p* < 0.0001), and missed the feeling of being understood by colleagues/employers (in EH 27 patients (48.2 %), in CH 27 patients (51.9 %), *p* = 0.039). In EH but not in CH significantly more patients with depression and/or anxiety reported that they had been less successful in their careers, had reduced earnings due to headaches, and that they felt less understood by their families (Table 2). In CH but not in EH significantly more patients with depression and/or anxiety avoided telling other people about their headaches, and the number of patients who lost more than ten days because of headache during the previous three months was higher in those with depression and/or anxiety; but the latter did not reach statistical significance. In both groups the interference with education and the feeling of being in control of the headache was independent of depression and/or anxiety (Table 2).

**Table 2:** Gender, headache diagnoses and burden of headache in patients with EH and CH with and without depression and/or anxiety. HADS scores ≥8 indicate presence of depression and/or anxiety, HADS scores <8 indicate absence of depression and/or anxiety

	EH (*n* = 232)				Statistics		CH (*n* = 160)				Statistics	
	Anxiety and/or depression						Anxiety and/or depression					
	Absent		Present				Absent		Present			
	Patients		Patients		Chi^2^	p	Patients		Patients		Chi^2^	p
	*n*	%	*n*	%			*n*	%	*n*	%		
Sex												
Missing data: 14						ns						ns
Female	112	83.6	80	87			35	63.6	70	72.2		
Male	22	16.4	12	13			20	36.4	27	27.8		
Headache diagnosis												
Missing data: 14												
Migraine	113	84.3	82	89.1		*ns	24	43.6	43	44.3		*ns
Tension-type headache	18	13.3	7	7.6		*ns	12	21.8	10	10.3		*ns
pMOH	-	-	-	-		-	19	34.5	41	42.3		*ns
Other headache	3	2.2	3	3.3		*ns	0	0	3	3.1		*ns
Quality of life												
Missing data: 15					40.8	0.001					32.6	<0.0001
Very good	34	25.4	11	12			17	30.9	5	5.2		
Good	87	64.9	39	42.4			23	41.8	26	27.1		
Neither good nor poor	12	9	30	32.6			13	23.6	35	36.5		
Poor	1	0.7	9	9.8			1	1.8	19	19.8		
Very poor	0	0	3	3.3			1	1.8	11	11.5		
Employment status												
Missing data: 31					10.0	0.002					17.0	<0.0001
Employed	116	89.9	64	73.6			46	88.5	51	54.8		
Unemployed/retired	13	10.1	23	26.4			6	11.5	42	45.2		
Lost days in preceding 3 months**												
Missing data: 56						ns						ns
0-5	41	32.3.	23	28.4			13	26.5	10	12.7		
6-10	21	16.5	14	17.3			3	6.1	3	3.8		
11-20	27	21.3	13	16			7	14.3	11	13.9		
>20	38	29.9	31	38.3			26	53.1	55	69.6		
Headache interfered with education												
Missing data: 20						ns						ns
Yes	38	29	36	39.6			20	37	45	46.9		
No	93	71	55	60.4			34	63	51	53.1		
Less successful in career due to HA												
Missing data: 22					7.3	0.007						ns
Yes	34	25.8	38	43.2			20	37	49	51		
No	98	74.2	50	56.8			34	63	47	49		
Reduced earnings due to HA												
Missing data: 57					9.1	0.003						ns
Yes	11	9.1	20	24.7			9	20	31	35.2		
No	110	90.9	61	75.3			36	80	57	64.8		
HA understood by colleagues/employer												
Missing data: 157					11.5	0.001					4.3	0.039
Yes	73	78.5	29	51.8			24	70.6	25	48.1		
No	20	21.5	27	48.2			10	29.4	27	51.9		
HA understood by family												
Missing data: 25					8.8	0.003						ns
Yes	128	96.2	74	85.1			49	90.7	76	81.7		
No	5	3.8	13	14.9			5	9.3	17	18.3		
Avoid telling people about HA												
Missing data: 19					7.8	0.05					4.9	0.028
Yes	33	24.8	38	42.7			19	34.5	51	53.1		
No	100	75.2	51	57.3			36	65.5	45	46.9		
Feeling of being in control of the headaches												
Missing data: 18						ns						ns
Yes	91	67.9	58	65.2			34	63	52	53.6		
No	43	32.1	31	34.8			20	37	45	46.4		

*compared to all remaining diagnoses, ** including lost workdays, lost housework days, workdays and housework days with productivity reduced to < 50 %, and lost days with leisure or family activities. *ns* not significant, *na* not applicable

These findings appeared even more pronounced using risk calculations (Table 3). Analysing all patients the presence of depression and/or anxiety increased the risk of CH (OR 2.5) and pMOH (OR 2.48), it markedly decreased quality of life and it increased burden. The risk of poor or very poor quality of life was almost 18-fold higher in patients with depression and/or anxiety compared to those without. The burden was higher for seven of the other nine variables with odds ratios ranging from 1.75 to 4.8 (Table 3). Assessing the impact of depression and/or anxiety separately for EH and CH the odds ratio for poor or very poor quality of life was 19.95 and 12.05, respectively, and the burden was increased in EH and CH in six and four of the other nine variables showing odds ratios of 2.2. to 4.5 and 2.15 to 6.3, respectively (Table 3).

**Table 3:** Odds ratios of having greater burden for presence versus absence of depression and/or anxiety

	All patients (*n* = 392)		EH (*n* = 232)		CH (*n* = 160)	
	OR	CI	OR	CI	OR	CI
Being male	1.1	0.67-1.80	0.76	0.36-1.63	0.68	0.33-1.37
Having chronic headache	2.57	1.68-3.93	na	na	na	na
Having migraine	1.35	0.87-2.10	0.66	0.29-1.47	1.03	0.53-2.00
Having pMOH	2.48	1.88-4.46	na	na	1.39	0.70-2.76
Having poor/very poor quality of life	17.84	5.42-58.70	19.95	2.55-156.33	12.05	2.75-52-72
Being unemployed/retired	4.8	2.7-8.5	3.21	1.52-6.76	6.31	2.46-16.22
Having ≥11 lost days in preceding 3 months	1.75	1.12-2.74	1.13	0.65-1.98	2.46	1.06-5.72
HA interfered with education	1.60	0.92-2.82	1.60	0.91-2.82	1.5	0.76-2.97
Less successful in career due do HA	2.19	1.43-3.37	2.19	1.23-3.89	1.77	0.90-3.51
Reduced earning due to HA	3.16	1.78-5.59	3.28	1.47-7.29	2.16	0.93-5.10
HA not understood by colleagues/employer	3.23-	1.85-5.64	3.40	1.65-6.99	2.59	1.04-6.48
HA not understood by family	3.54	1.68-7.48	4.50	1.54-13.12	2.19	0.76-6.33
Avoid telling people about HA	2.43	1.58-3.73	2.26	1.27-4.02	2.15	1.08-4.26
Feeling of being in control of the headaches	0.73	0.48-1.11	0.88	0.50-1.56	0.68	0.34-1.34

*OR* odds ratio, *CI* 95 % confidence interval, *na* not applicable

Patients with CH consulted general practitioners significantly more often than patients with EH (61/39.1 % vs. 65/29.1 %; *p* = 0.043), consultations of other health care professionals did not differ between patients with EH and CH. Depression and/or anxiety had no impact on headache-related health care consultations (headache specialist, general practitioner, hospital emergency room, nurse, physical therapist) and examinations (magnetic resonance imaging, computer tomography, X-ray, eye test, blood tests) during the previous 12 months in EH and CH. The impact of depression and/or anxiety on the use of five classes of standard prophylactic medications was minimal. In EH but not in CH patients with depression and/or anxiety had significantly more contraindications than those without (EH without depression and/or anxiety 29 patients (23.2 %), EH with depression and/or anxiety 31 (36.5 %), *p* = 0.037; CH without depression and/or anxiety 12 patients (22.6 %), CH with depression and/or anxiety 18 (20.9 %), *p* > 0.05. Neither in EH nor in CH ever-before use of prophylactics, adverse effects, use for at least three months, intake of an adequate dose and decrease of headache frequency by at least 50 % was statistically associated with depression and/or anxiety (Table 4).

**Table 4:** Summarized data for five classes of standard prophylactic medications (betablocker, flunarizine, valproate, topiramate, amitriptyline) in patients with EH and CH with and without depression and/or anxiety, differentiating “none” or “one and more prophylactics”. HADS scores ≥8 indicate presence of depression and/or anxiety, HADS scores <8 indicate absence of depression and/or anxiety

	EH (*n* = 232)						CH (*n* = 160)					
	Anxiety and/or depression				Statistics		Anxiety and/or depression				Statistics	
	Absent		Present				Absent		Present			
	Patients		Patients		Chi^2^	p	Patients		Patients		Chi^2^	p
	*n*	%	*n*	%			*n*	%	*n*	%		
Ever-before use												
Missing data: 27												
None	59	45.7	40	44.9			27	50	39	41.9		
One ore more prophylactics	70	54.3	49	55.1			27	50	54	58.1		
Contraindications												
Missing data: 43					4.4	0.037						ns
None	96	76.8	54	63.5			41	77.4	68	79.1		
One or more	29	23.2	31	36.5			12	22.6	18	20.9		
Adverse effects												
Missing data: 25						ns						ns
None	43	68.3	30	69.8			21	87.5	34	66.7		
Caused by one or more prophylactics	20	31.7	13	30.2			3	12.5	17	33.3		
Use for ≥ 3 months												
Missing data: 19						ns						ns
None	17	26.6	7	14.9			5	19.2	9	18		
One or more prophylactics	47	73.4	40	85.1			21	80.8	41	82		
Intake of an adequate dose												
Missing data: 23						ns						ns
None	5	7.9	6	13.6			4	15.4	9	18		
One or more prophylactics	58	92.1	38	86.4			22	84.6	41	82		
Decrease in HA frequency by ≥ 50 %												
Missing data: 33						ns						ns
None	28	50.9	25	58.1			13	52	35	70		
Caused by one or more prophylactics	27	49.1	18	41.9			12	48	15	30		

*ns* not significant

## Discussion

This post-hoc analysis of a prospective, cross-sectional multicentre study in eight Austrian headache centres showed a significantly higher prevalence of anxiety and/or depression in CH (64 %) than EH (41 %). Depression and anxiety had a marked impact on quality of life and increased the burden in patients with EH and CH as well as in the entire group of patients. Patients with depression and/or anxiety had statistically a higher risk of CH and pMOH. In contrast, diagnostic and therapeutic management of headache was unrelated to the presence of depression and/or anxiety with only one single exception, i.e. an increased risk of contraindications against standard prophylactic medications in EH.

These findings replicated in a clinic-based population well-known findings that patients with CH suffer from depression and/or anxiety more often than patients with EH [1, 9, 23–26], including suicidal attempts as shown in the literature [27]. Based on the concept of stagnation, Innamorati et al. [28, 29] showed that patients with medication overuse headache are burdened with a combination of psychological, behavioural and physical symptoms, and that in chronic migraine stagnation, characterized by feelings of obstruction of body, emotions and behaviour, is associated with depression. The impact of depression and anxiety on burden is much less known, however, and has been examined in three studies only [12–14]. A study in patients with chronic migraine and medication overuse found that depressive symptoms in these patients were associated with higher disability and lower quality of life [12]. Patients with posttraumatic stress disorder and migraine were more likely to be in the low poverty index and less likely to work for pay than patients with migraine alone, their work quality was cut 2.5-fold greater than in patients with migraine alone, and their difficulties getting along with social life were 2-fold greater than in patients with migraine alone [13].

Recurrent or chronic headache and psychiatric comorbidities interact in a complex manner. Some longitudinal studies found clues for a bidirectional association: In adolescents anxiety and depression were associated with recurrent migraine (not tension-type headache) after four years [30], a higher migraine frequency was associated with higher depression and anxiety scores [31], depression and anxiety were significant risk factors for the chronification of migraine [12, 32, 33]. The risk of having a major depression in patients with migraine was as high as the risk for getting migraine in patients with major depression, and the risk for major depression was higher in persons with migraine compared to persons without migraine [33]. A recent study found an increased migraine frequency in patients with anxiety or depression [14]. Our study was a cross-sectional study, therefore odds ratios have to be interpreted cautiously.

Our data showed that the impact of depression and anxiety on daily activities is high in patients with CH. Over 80 % of the patients with CH and depression and/or anxiety lost more than ten days of productivity, family, social and leisure activities during the previous three months. This may increase their risk of losing the job, thus contributing to the lower socioeconomic status of patients with chronic pain [5]. In contrast, the numbers of lost days were comparable in the other study groups, i.e. EH without and with depression and/or anxiety and CH without depression and/or anxiety. Markedly more patients in our study reported that their headaches interfered with education, careers and earnings than in the study of Steiner et al. [10], and these reports were even higher in patients with depression and/or anxiety, although not statistically significant. There is a substantial impact on education, career and earnings in the patients’ perception. Lower education levels and lower socioeconomic status were associated with an increased risk of getting migraine [34], and patients with chronic migraine were less likely to be working for pay than patients with episodic migraine [35]. So far, the impact of EH, CH and depression and/or anxiety on the employment status has not been studied.

Furthermore, social support was affected by the presence of anxiety and/or depression resulting in increased risks of feeling less understood by the family, employer and colleagues as well as an increased risk of avoiding to tell other people about the headache. This was true for the entire group of patients as well as for those with EH and CH, with one exception. The difference for the family’s understanding on ones headaches did not reach the level of statistical significance in CH. In contrast, D’Amico et al. [36] found only little evidence for the impact of perceived social support on chronic migraine.

In accordance with other studies [25, 37] patients with CH consulted general practitioners more often. In the present subgroup analysis we did not find differences in health care use and technical examinations between patients with and without depression and/or anxiety. But the numbers of patients in each group were relatively small, so we cannot draw a definite conclusion with regards to the influence of depression and anxiety. A recent study [38] found in-hospital stays six times, outpatient visits four times and emergency department visits three times as much in migraine patients with compared to those without psychiatric comorbidities. In addition the patients with psychiatric comorbidities underwent computer tomography and magnetic resonance imaging of the brain more often.

As in the main study the ever-before use of prophylactic medication was low, but in contrast to the main study the ever-before use did not differ between patients with EH and CH [11]. This may be attributed to the smaller patient number in the present analysis. Moreover the overall low use of prophylactic drugs may be caused by patient or physician related factors: patients may deny taking prophylactic drugs, neurologists may try non-drug prophylaxis first, or especially physicians who are not specialized in headache treatment may not think of prophylactic drugs. We did not ask the treating neurologists and patients for the reasons of not prescribing or taking prophylactics, and we are not aware of other studies dealing with this issue. Interestingly, betablocker and flunarizine, although having the potential of triggering or worsening depression, had ever been used by a substantial number of patients with depression and/or anxiety. The neurologists assessed possible contraindications based on the patient’s history and on their own discretion. For clarity and comparability for each drug the main contraindications according to the prescribing information were listed in the questionnaire. The prescription pattern in this study also reflects the treatment of headache patients in neurological offices, as we included patients who came for a first-time visit to one of the headache centres as well as patients who came for a control visit. Our findings emphasize that the choice of prophylactic drugs needs to be improved, especially with regards to depression and anxiety. Adverse effects, duration of prophylactic treatment, intake of an adequate dose and decrease in headache frequency by ≥ 50 % did not differ between patients with and without anxiety and/or depression, but patient numbers in the groups were too small do draw definite conclusions. Given the impact of depression and anxiety on recurrent and chronic headache further studies on therapeutic impact are urgently needed. To the best of our knowledge no previous study addressed this topic up till now.

Limitations of this study are the post-hoc analysis and relatively small patient numbers in the subgroups of some items. As this was an exploratory analysis, we did not correct for multiple testing. We did not analyse the dataset differentiated by migraine, tension-type headache, pMOH and other headaches or differentiated by anxiety alone, depression alone or anxiety and depression, because the subgroups would have been too small. The HADS is a validated and widely used questionnaire for screening for depression and anxiety, but it does not allow a clinical diagnosis of depression or anxiety, and patients in this study did not undergo a psychiatric interview. Finally, we must bear in mind that comorbidity rates in clinic-based studies may be higher than in population-based studies because of an increased vigilance or selection bias.

Strengths of the study are the large clinic-based sample from eight headache centres with high participation rates, and the use of validated questionnaires for establishing headache diagnoses and for assessing depression and anxiety.

## Conclusion

This is one of the first studies looking at the impact of depression and anxiety on EH and CH in a clinic-based patient sample. Patients with CH suffer from depression and/or anxiety significantly more often than patients with EH. More importantly, the quality of life is deceased and the burden is further increased by depression and/or anxiety in patients with EH and CH. In addition, treatment with prophylactic medications is sub-optimal in patients with depression and/or anxiety. In particular for these patients a comprehensive treatment approach including pharmacological and non-pharmacological treatments (such as behavioural therapy, relaxation training, coping strategies) is necessary in order to decrease disability on the personal, social and occupational level.
